# Association between a new dietary protein quality index and micronutrient intake adequacy: a cross-sectional study in a young adult Spanish Mediterranean cohort

**DOI:** 10.1007/s00394-022-02991-z

**Published:** 2022-09-10

**Authors:** Víctor de la O, Itziar Zazpe, Carmen de la Fuente-Arrillaga, Susana Santiago, Leticia Goni, Miguel Ángel Martínez-González, Miguel Ruiz-Canela

**Affiliations:** 1 Department of Preventive Medicine and Public Health, Campus Universitario, School of Medicine, University of Navarra, 31008 Pamplona, Spain; 2 Department of Nutrition and Food Sciences and Physiology, Campus Universitario, School of Pharmacy and Nutrition, University of Navarra, 31008 Pamplona, Spain; 3 CIBER Fisiopatología de La Obesidad Y Nutrición (CIBEROBN), Instituto de Salud Carlos III (ISCIII), Madrid, Spain; 4 Navarra Institute for Health Research (IdiSNA), Pamplona, Spain; 5 Department of Nutrition, Harvard TH Chan School of Public Health, Boston, MA USA; 6 Cardiometabolic Nutrition Group, IMDEA Food, CEI UAM + CSIC, Madrid, Spain

**Keywords:** Protein source quality, Micronutrient intake adequacy, Mediterranean diet, Vitamins, Minerals, Cohort

## Abstract

**Purpose:**

There is no evidence of a dietary index that measures not only the quantity but also the quality of protein. The aim is to investigate the association between a new dietary protein quality index (PQI) and micronutrient intake adequacy in a Mediterranean cohort.

**Design:**

We assessed 17,535 participants’ diet at baseline using a semi-quantitative FFQ. The PQI was calculated according to the ratio of protein (g/d) sources: [fish, seafood, lean meat, pulses, eggs, nuts, low-fat dairy, and whole grains]/[red and ultra-processed meats, whole-fat or semi-skimmed dairy, potatoes and refined grains]. Participants were classified into quintiles of PQI. We evaluated the intakes of Fe, Cr, I, K, Mg, Ca, P, Na, Se, Zn and vitamins A, B1, B2, B3, B6, B12, C, E and folic acid. Micronutrient adequacy was evaluated using DRIs. Logistic regression analysis was used to assess the micronutrient adequacy according to quintiles of PQI.

**Results:**

In this cross-sectional analysis, a total of 24.2% and 4.3% participants did not to meet DRIs in ≥ 4 and ≥ 8 micronutrients, respectively. The odds of failing to meet ≥ 4 and ≥ 8 DRI were lower in participants in the highest quintile of protein quality (OR = 0.22; IC 95% = 0.18, 0.26; P-trend < 0.001; and OR = 0.08; IC 95% = 0.05, 0.14; P-trend < 0.001, respectively) as compared to participants in the lowest quintile.

**Conclusion:**

Higher PQI was found to be strongly associated with better micronutrient intake adequacy in this Mediterranean cohort. The promotion of high-quality protein intake may be helpful for a more adequate intake of micronutrients. The odds of failing to meet certain numbers of DRIs were lower rather than saying lower risk.

**Supplementary Information:**

The online version contains supplementary material available at 10.1007/s00394-022-02991-z.

## Introduction

Dietary proteins play an important role in growth, weight management and satiety [1], metabolic and renal function [2], prevention of muscle loss and management of sarcopenia on healthy aging and bone health [3]. An adequate protein intake is also key to build healthier diets adherence [4]. In terms of quantity, for adults aged > 19 years the Recommended Dietary Allowance (RDA) for proteins is 0.8 g of good quality protein per kilogram of body weight per day, representing 10–35% of total daily energy intake [5, 6]. This recommended amount is intended to maintain relative energy balance with amino acid oxidation and urea excretion, and nitrogen/protein balance [6].

Protein quality definition has been traditionally based on the quantity and their digestibility and the variety of amino acids. In the early nineties, the Food and Drug Administration, FAO and WHO adopted the Protein Digestibility-Corrected Amino Acid Score which defined protein quality according to human amino acid needs and human’s ability to digest [7]. Afterward, the Digestible Indispensable Amino Acid Score (DIAAS) was considered a more accurate protein quality method because it provides the proportion of the amino acid absorbed at the end of the small intestine [8]. The protein quality is based on the distribution of amino acids contained in the protein and in what amount the limiting amino acids (amino acids present in extremely low amounts in a food in relation to dietary needs) are available [9]. According to these criteria to define protein quality, animal protein has been traditionally classified as better-quality protein sources as compared to vegetable proteins because of their digestibility and their amino acid composition [10].

However, traditional scores of protein quality have some limitations [8, 11]: they do not take into account the net effects on human and environmental health, their limited representation of commonly consumed plant-based foods within the assessment framework [12], and insufficient awareness of the digestibility of commonly consumed heat-treated and processed plant-based foods [12]. DIAAS scores are intended to measure food quality, but do not take into account the food's net effects on human health and environmental impact. The FAO emphasized in its latest report on healthy and sustainable dietary guidelines, that a diet with lower environmental impacts could have associated health benefits [13]. Changing animal to plant proteins would reduce greenhouse gasses emissions, land use, and saturated fat intake [13], and replacing animal protein to legumes would also increase the intake of folic acid, high-quality carbohydrates (dietary fiber) and several other nutrients. Therefore, there is growing evidence that highlights the need to redefine the concept of protein quality, taking quality into account at this point [14]. In fact, it is suggested that with a proper combination of food sources, plant proteins can provide greater reductions in saturated fat intake and greater increases in fiber, vitamin A, vitamin C, folate, Mg, and K compared to animal sources [15]. However, to the best of our knowledge, no specific score, considering this broader definition of protein quality has been proposed in the context of epidemiological studies.

An adequate protein intake should be promoted to achieve the required micronutrient intake [16]. Commonly consumed food sources of protein are more than just protein but also significant sources of essential nutrients. Sources of dietary protein frequently contribute substantially to intakes of nutrients, such as Ca, vitamin D, K, dietary fiber, Fe, and folic acid, which have been identified as nutrients of “concern” (i.e., intakes are often lower than recommended) [17]. Current evidence suggests that the consumption of a variety of protein food sources, both animal and plant-based, is important to meet nutrient recommendations [17, 18]. Two previous studies have shown that greater consumption of protein-containing foods increases micronutrient adequacy, but they did not explore the relevance of protein quality [19, 20]. In previous investigations within the *Seguimiento Universidad de Navarra* (SUN) cohort and the PREvención con DIeta MEDiterránea (PREDIMED) study, we showed an association between both the carbohydrate quality index and the fat quality index with better micronutrient adequacy [21–23]. However, as far as we know, no previous study has assessed the association between a protein source quality index and micronutrient intake.

Our aim was, first, to define a new score for assessing not only the quantity but also the quality of protein intake that takes into account the amino acid profile, the association with health and the environmental impact; and second, to investigate the cross-sectional association between this score and the intake adequacy of 19 micronutrients in the SUN cohort, a Mediterranean cohort of young adults in Spain.

## Subjects and methods

### Study design and population

The SUN Project is a dynamic and multipurpose prospective cohort study conducted in Spain among university graduates since December 1999. This selection of highly educated participants corresponds to the approach known as restriction in epidemiology and it was applied to control for confounding by socioeconomic status [24, 25]. We are inviting to participate all Spanish alumni of the University of Navarra and several other professional collectives with a university degree. We select only those university graduates who are willing to commit themselves for returning questionnaires every 2 years. This cohort assesses the associations between diet and lifestyle and the occurrence of several diseases and chronic conditions. The recruitment is permanently open, and participants are followed-up biennially using questionnaires distributed by post or electronic mail. Overall, most participants are young adults (median age: 35 years, 82% younger than 50 years). The majority are women (61%), married (50%) or single (46%), and are graduated in a health-related profession (55%). A baseline questionnaire collects information related to lifestyle, medical history, socio-demographics, anthropometric, and several diet variables. More detailed information about this cohort has been previously described elsewhere [24].

**Fig. 1:**
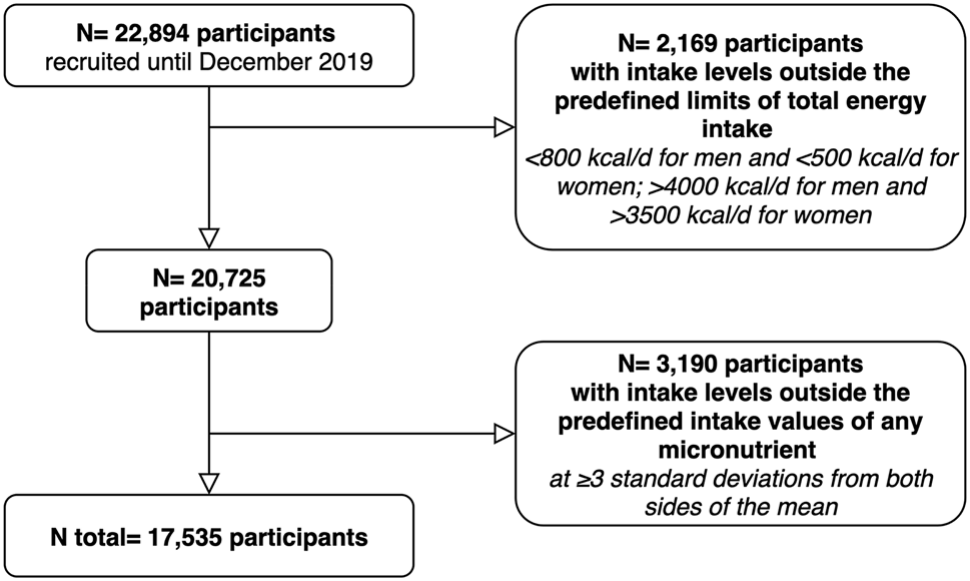
Flowchart of participants in the SUN Project, 1999–2019

In this study, we used a cross-sectional design to assess the association between the Protein source Quality Index (PQI) and micronutrient adequation at baseline. As of December 2019, the dataset collected a total of 22,894 participants who had answered the baseline questionnaire. We excluded 2169 individuals with intake levels outside predefined limits of total energy intake: < 800 kcal/d for men and < 500 kcal/d for women; > 4000 kcal/d for men and > 3500 kcal/d for women [26]. Finally, we excluded 3190 participants whose intake levels were outside predefined intake values of any micronutrient (≥ 3 standard deviations from both sides of the mean). Therefore, the final sample comprised 17,535 participants (Fig. 1).

### Ethics

All participants received at entry to the SUN project a detailed written information and gave their permission to participate in the study. Voluntary completion of the baseline self-administrated questionnaire was considered to imply informed consent. This study was conducted according to the Declaration of Helsinki guidelines, and it was approved by the Research Ethics Committee of the University of Navarra. The SUN project is registered at clinicaltrials.gov as NCT02669602.

### Exposure assessment: the dietary Protein source Quality Index

At baseline, food intake was assessed using a 136-item semi-quantitative food frequency questionnaire (FFQ), considering the consumption frequency over the past year. This FFQ was validated in Spain and subsequently re-evaluated [24, 27, 28]. The consumption of foods was asked at each item of the FFQ with a single frequency response ranging from “never or almost never” to “ ≥ 6 times a day”. Daily consumption of every food item was estimated by multiplying its typical portion size with its frequency of consumption using an ad hoc computer program specifically developed for this aim [29, 30].

The following criteria were applied to define the dietary PQI [5, 30–32]. First, we selected the main protein sources according to their contribution of foods in total protein intake among all participants of the SUN cohort (*n* = 22,894 recruited at baseline). We chose the following 12 food groups: red and processed meats, full-fat dairy, whole grains, potatoes and refined grains, lean meats, lean fish, reduced-fat dairy, pulses, fatty fish, eggs, seafood and finally nuts. All these food groups contributed to 81.84% of total protein intake in our population (Supplemental Table 1).

Second, we assessed the content of essential amino acids for each food source, and we determined the total quantity essential amino acid per 100 g of each food groups (Supplemental Table 2) using the German “Food Composition and Nutrition Tables (2008)” [32] and the USDA database [33]. Thus, we calculated essential amino acid content for each food group: histidine (His), isoleucine, (Ile) leucine (Leu), lysine (Lys), methionine (Met), phenylalanine (Phe), threonine, (Thr) tryptophan (Trp), and valine (Val). In this step we confirmed that the 12 food groups previously selected were the main contributors of essential amino acids.

Third, we classified protein-rich sources as healthy or unhealthy in accordance with Harvard and the American recommendations dietary guidelines [5, 34–36], and its climate impact measured as CO_2_ equivalent per kg edible weight according to the evidence [13]. Thus, the food group received one point for each one of the following criteria: high protein intake, high content of essential amino acids, consumption recommended for health reasons, and low climate impact with ≤ 4 kg CO_2_ equivalent per kg edible weight. Thus, to create the PQI, all the partial scores were added, and the final range was between 0 and 4. All foods or food groups with a score of 4 were assigned to the numerator (best quality) and those with ≤ 3 or fewer points to the denominator (Supplemental Table 3). Full-fat dairy products were located as unhealthy food groups due to its association with saturated fats and the risk of cardiovascular disease [37]. Red meats were also located as unhealthy food groups due to its consumption has been linked to major chronic diseases, such as diabetes, coronary heart disease, heart failure, stroke and cancer at various sites, and mortality [38]. Therefore, the dietary PQI was calculated using the following ratio: PQI = [proteins (g/d) from fish (lean and fatty) + seafood + lean meat + pulses + eggs + nuts + (low) fat dairy products + whole grains]/[proteins (g/d) from red and (ultra) processed meats + (whole and skimmed)fat dairy products + potatoes and refined grains].

### Outcome assessment: micronutrient adequacy

In this study we assessed the following micronutrients: Fe, Cr, I, K, Mg, Ca, P, Na, Se, Zn and vitamins A, B1, B2, B3, B6, B12, C, E and folic acid. Baseline intake of these micronutrients was calculated using the FFQ and considering both the intake from foods and from dietary supplements. Micronutrient intakes were calculated as frequency multiplied by nutrient composition of specified portion size for each food item. It was used a computer-based program on available information in Spanish food composition tables [29, 30]. We used the Dietary References Intakes (DRI) values defined as quantitative estimates of nutrient intakes to be used for assessing and planning diets for healthy people [5, 14]. The DRI proposed by Institute of Medicine included four different values: Recommended Dietary Allowance (RDA), Adequate Intake (AI) values for nutrients having undetermined RDA, Estimated Average Requirement (EAR), and finally, Tolerable Upper Level values (UL).

We used the probabilistic approach to calculate the probability of intake inadequacy of all micronutrients with EAR values, as follows: z score = (estimated nutrient intake − EAR)/SD of the EAR. The *z* score corresponded to an estimated probability of inadequacy according to the normal distribution. This *z* score was not calculated for K and Cr because these micronutrients have AI values, but not EAR values [39]. Fe intake followed a skewed distribution, and for this reason we first log transformed its values.

### Other co-variates

The baseline questionnaire in the SUN cohort includes several questions to collect sociodemographic data, lifestyle and health-related habits, self-reported anthropometric measures, family and personal medical history, and medication use. Physical activity information was gathered using the validated Spanish version of the Nurses’ Health Study and the Health Professionals Follow-up Study questionnaires [40]. Two subsamples of the SUN cohort were used to assess accuracy of self-reported weight and height [41] and physical activity [42]. We also used the Mediterranean Diet Score (MDS) [43] to assess the adherence to a Mediterranean diet pattern.

### Statistical analyses

We calculated age and sex-adjusted baseline characteristics of participants using the Inverse Probability Weighting method. These variables were expressed as percentages and mean (SD) for categorical and continuous variables, respectively.

We used crude and multivariable logistic regressions models to assess the association between quintiles or deciles of the PQI and micronutrient intake adequacy at baseline using the EAR cut-off points and the probabilistic approach. We defined two different outcomes, according to two different cut-off points: ≥ 4 and ≥ 8 micronutrients with unmet DRI ≈25% and 50% respectively of the 19 micronutrients. We calculated odds ratios (ORs) of unmet micronutrient goals (≥ 4 or ≥ 8 micronutrients) with their 95% confidence interval (CIs) and we considered always the lowest quintile of the PQI as the reference category. Additionally, tests of linear trend across successive categories of PQI were conducted assigning the median value to each category and treating the variables as continuous.

We ran three multivariable-adjusted models: model 1 adjusted for age (continuous), sex and education level (graduate, master, and doctorate); model 2 additionally adjusted for energy intake (continuous); and model 3 additionally adjusted for the MDS (continuous), BMI (continuous), physical activity (continuous, MET-h/week), and smoking status (no smoking, former, current < 15 cig/d and current ≥ 15 cig/d). We did not adjust by socioeconomic status, because the baseline questionnaire does not collect this information.

We created a new variable combining the exposure to categories of PQI (Q1, merged Q2-Q4 and Q5) and adherence to MDS (in 2 groups categorized by the median) to evaluate the combined effect of the quality protein intake and Mediterranean diet adherence on unmet EAR for at least 50% of nutrients. We used as reference category the group of participants with best PQI and MDS. The likelihood ratio test was used to test the statistical significance of the interaction term between the MedDiet index categories and the PQI categories. As supplementary analysis, Student *t* tests were run to compare mean differences of inadequate minerals and vitamins intake between extreme quintiles of the PQI according to adherence to MDS. For this analysis we adjusted for total energy intake using the residual method [44].

Pearson’s correlation was applied to assess the association between each of the 19 micronutrients and animal protein (dairy protein, eggs, meat, fish, and processed and ultra-processed foods of animal origin), plant protein (vegetables, fruits, nuts, legumes, cereals, and soft drinks made with vegetable sources), and total protein (animal + plan protein). The total animal and plan protein was calculated using 85 and 47 items of FFQ, respectively. Pearson’s correlation was applied to assess the association of total protein, animal protein and plant protein with each of the 19 micronutrients.

We applied 4 sensitivity analyses. First, we analyzed the PQI adjusted for energy intake with the residual method; second, we analyze a modified PQI (Healthy Plate source Quality Index, HPPQI) based on the model for healthy proteins proposed in the Harvard’s Healthy Eating Plate [35]; third, no-excluding participants with implausible intakes; and fourth, applying different exclusions energy intakes criteria according to percentiles (P95;  P99). For the HPPQI was calculated with the following ratio: HPPQI = [proteins (g/d) from seafood + poultry + pulses + nuts] / [proteins (g/d) from red and processed meats + cheese] [45].

Analyses were performed with STATA version 14.1 (STATA Corp., TX, USA). All *P* values are two-tailed and statistical significance was set at the conventional cut-off of *P* < 0.05.

## Results

Age and sex-adjusted baseline characteristics of the 17,535 participants according to quintiles of the dietary PQI are summarized in Table 1. Participants with a higher PQI score were more likely to be single, former smokers, physically active, to follow a special diet, and to have higher baseline prevalence of hypertension, hypertriglyceridemia, and hypercholesterolemia; whereas they were less likely to snack between meals. Besides, those participants with higher PQI had also higher adherence to the MDS and higher consumption of fruits, nuts, fish, vegetables, lean meats, whole cereals and grains, reduced-fat dairy products and pulses; and a lower consumption of eggs, red and ultra-processed meats, and food products, full-fat dairy products and sugar-sweetened beverages (Table 1).

**Table 1:** Age and sex-adjusted baseline characteristics of the participants in the SUN cohort according to quartiles of the PQI score (1999 – 2017)^1^

Characteristics	Protein source quality index				
	Q1 (lowest quality)	Q2	Q3	Q4	Q5 (highest quality)
Score min to max (median)	0 to < 0.5 (0.4)	0.5 to < 0.7 (0.6)	0.7 to < 0.9 (0.8)	0.9 to < 1.3 (1)	1.3 to 41.1 (1.8)
N	3507	3507	3507	3507	3507
Women (%)	60.4	59.8	59.5	59.7	61.4
Marital status (%)					
Single	44.8	44	44	43.6	47.6
Married	50.5	50.8	50.5	50.4	46.4
Widowed	1	0.9	0.9	0.8	0.8
Separated	1.7	2.4	2.4	2.8	3
Education level (y)	5.1 ± 1.6	5.1 ± 1.5	5 ± 1.5	5.1 ± 1.5	5 ± 1.5
Tobacco habit (%)					
No smoking	48.1	48.6	46.2	48.7	49
Former	27.6	28.2	28.5	29	31.9
Current < 15 cig/d	12.5	11.7	13.8	12.8	10.4
Current ≥ 15 cig/d	11.3	10.7	10.7	8.6	7.9
Physical activity (METs-h/week)	18.8 ± 20.4	19.6 ± 20.4	21.2 ± 22.1	22.5 ± 23.3	24.8 ± 25.3
BMI (kg/m^2^)	23.1 ± 3.4	23.4 ± 3.4	23.6 ± 3.5	23.8 ± 3.6	23.8 ± 3.6
Sitting (h/d)	5.4 ± 2.1	5.3 ± 2	5.3 ± 2	5.2 ± 2	5.2 ± 2.1
Snacking between meals (%)	35.1	35.8	33.7	33.3	29.9
Follow a special diets (%)	3.9	4.3	5.4	8.3	16.3
Napping (%)	53	53.9	56.6	56.1	54.8
Prevalent diseases (%)					
Hypertension	8.7	9.3	11.2	11.5	13.5
Hypertriglyceridemia	4.6	5.1	6.7	8.1	9.7
Hypercholesterolemia	12.1	14.1	16	18.9	23.7
Cancer	2.8	2.5	2.3	2.5	2.5
Diet					
Supplement use (%)	2.5	2.7	2.8	3.1	3.2
Mediterranean diet score (MDS) [38]	2.7 ± 1.4	3.3 ± 1.5	3.8 ± 1.5	4.2 ± 1.5	4.9 ± 1.5
Mediterranean Diet Adherence Screener (MEDAS) [50]	4.8 ± 1.6	5.5 ± 1.6	5.9 ± 1.6	6.4 ± 1.6	7.3 ± 1.7
Fruits (g/d)	272.2 ± 213.9	298.2 ± 224.5	320.8 ± 242.2	342.3 ± 263.2	380.1 ± 273.2
Nuts (g/d)	5.1 ± 7.2	6.3 ± 8.8	7.1 ± 10	7.7 ± 10.9	9 ± 12.7
Fish (g/d)	60.2 ± 32.4	82.6 ± 39.5	96.7 ± 46.6	110 ± 52.9	119.4 ± 59.5
Eggs (g/d)	22.1 ± 13.2	24.7 ± 14.8	23.9 ± 14.8	23.5 ± 17.3	21.5 ± 17.7
Vegetables (g/d)	418.5 ± 238.5	467.4 ± 253	496.7 ± 259.1	526.5 ± 274.2	574.1 ± 297.2
Meats (g/d)	190.1 ± 76	186.3 ± 73	182.4 ± 72.4	170 ± 70.4	141.3 ± 70.9
Lean meat (g/d)	29 ± 19.3	41.1 ± 25.9	48 ± 30.1	53 ± 34.4	60.2 ± 41.9
Red and ultra-processed meat (g/d)	161.1 ± 67.5	145.2 ± 58.5	134.3 ± 54.9	117.1 ± 49.2	81.1 ± 43.8
Cereals and grains (g/d)	115.1 ± 76.9	104.6 ± 65.8	96.3 ± 61.9	89.8 ± 58.1	81.8 ± 55.8
Whole cereals and grains (g/d)	0.3 ± 0.9	0.5 ± 1.5	0.8 ± 2	1.2 ± 2.4	2 ± 3.2
Dairy products (g/d)	408.9 ± 211.5	370.9 ± 191.8	339.2 ± 187.7	326.5 ± 191.3	340.2 ± 206.5
Reduced-fat dairy products (g/d)	15.2 ± 44.7	35.3 ± 76.7	70.6 ± 120.1	129.1 ± 164.7	234.1 ± 207.4
Full-fat dairy products (g/d)	362.5 ± 209.2	303.2 ± 192.6	238 ± 181.8	167.5 ± 160.6	79.3 ± 114.1
Pulses (g/d)	18.1 ± 10.2	20.7 ± 11.2	22.5 ± 15.1	24.3 ± 18.1	26.6 ± 25.4
Ultra-processed food (g/d)	312.3 ± 190.6	301.5 ± 175.8	294.6 ± 177.7	286.7 ± 180.1	248.2 ± 183.8
Olive oil (g/d)	18.6 ± 15.8	18.7 ± 15.2	18.1 ± 14.4	18.1 ± 14.4	17.6 ± 14
Sugar-sweetened beverages (g/d)	50.2 ± 98.4	44.1 ± 80.8	40.1 ± 77.6	35.8 ± 70.8	25.7 ± 66.8

^1^ Means ± SD are shown unless otherwise stated. Table adjusted for age and sex by the Inverse Probability Weighting method

BMI, body mass index; MET, metabolic equivalent of task; Q, quintile

Table 2 shows age and sex-adjusted nutritional values of the study participants across the quintiles of the PQI. Participants with higher PQI had a lower intake of sugar, total fat, SFAs, cholesterol, and alcohol, whereas they presented slightly higher intake of MUFA and fiber. The intake of several micronutrients, including Ca, K, Mg, Zn, and vitamins A, B6, B12, C, D, E, and folic acid increased within the increasing quintiles of PQI, while Na intake was lower in the highest quintile**.** The contribution of different food groups on baseline essential amino acids is shown in Supplemental Table 2. Food groups were ordered according to their total amino acid content. Pulses, fish (fat and lean), seafood and meat (lean and red) were the main sources of amino acids.

**Table 2:** Age and sex-adjusted baseline nutritional values according to quintiles of the PQI in participants of the SUN cohort (1999 – 2017)^1^

Characteristics	Protein source Quality Index				
	Q1 (lowest quality)	Q2	Q3	Q4	Q5 (highest quality)
Score min to max (median)	0 to < 0.5 (0.4)	0.5 to < 0.7 (0.6)	0.7 to < 0.9 (0.8)	0.9 to < 1.3 (1)	1.3 to 41.1 (1.8)
*N*	3507	3507	3507	3507	3507
Energy and macronutrients					
Total energy (kcal/d)	2441 ± 575.7	2390 ± 572.3	2310 ± 581.2	2223 ± 571.5	2002 ± 540.8
Carbohydrate intake (% of TEI)	43 ± 7.1	42.7 ± 6.7	42.4 ± 6.9	42.8 ± 7.2	44.2 ± 7.8
Sugar (% of TEI)	2.5 ± 2.6	2.3 ± 2.5	2.1 ± 2.5	1.9 ± 2.4	1.6 ± 2.4
Protein intake (% of TEI)	16.8 ± 2.6	17.5 ± 2.6	18.1 ± 2.8	18.6 ± 3.1	19.6 ± 3.6
Animal protein^2^ (% of TEI)	12.6 ± 2.9	13.1 ± 2.8	13.5 ± 3	13.8 ± 3.3	14.1 ± 3.9
Plant protein^3^ (% of TEI)	4.3 ± 1.1	4.5 ± 1.1	4.7 ± 1.1	4.9 ± 1.3	5.5 ± 1.6
VAL (g/d)	6.1 ± 1.5	6.2 ± 1.5	6.1 ± 1.5	6 ± 1.6	5.6 ± 1.5
LEU (g/d)	8.9 ± 2.2	9 ± 2.2	8.9 ± 2.3	8.8 ± 2.3	8.2 ± 2.2
ILE (g/d)	5.5 ± 1.4	5.6 ± 1.4	5.6 ± 1.4	5.5 ± 1.5	5.1 ± 1.4
LYS (g/d)	8.5 ± 2.3	8.7 ± 2.3	8.7 ± 2.3	8.6 ± 2.4	7.9 ± 2.3
MET (g/d)	2.6 ± 0.7	2.6 ± 0.7	2.6 ± 0.7	2.6 ± 0.7	2.4 ± 0.7
PHE (g/d)	4.9 ± 1.2	4.9 ± 1.2	4.9 ± 1.2	4.8 ± 1.2	4.4 ± 1.2
THR (g/d)	4.8 ± 1.2	4.9 ± 1.2	4.9 ± 1.3	4.8 ± 1.3	4.5 ± 1.2
TRP (g/d)	1.3 ± 0.3	1.3 ± 0.3	1.3 ± 0.3	1.2 ± 0.3	1.1 ± 0.3
HIS (g/d)	3.1 ± 0.8	3.1 ± 0.8	3.1 ± 0.8	3 ± 0.8	2.7 ± 0.8
Fat intake (% of TEI)	38.3 ± 6.2	37.9 ± 5.8	37.3 ± 5.8	36.3 ± 6.1	34 ± 6.6
SFAs intake	13.8 ± 3	13.4 ± 2.7	12.8 ± 2.7	12.1 ± 2.8	10.4 ± 2.7
MUFAs intake	16.2 ± 3.5	16.2 ± 3.5	16 ± 3.4	15.7 ± 3.6	15 ± 3.9
PUFAs intake	5.1 ± 1.5	5.2 ± 1.4	5.2 ± 1.4	5.2 ± 1.4	5 ± 1.4
Cholesterol (mg/d)	411.3 ± 137.1	428.5 ± 136.3	422.7 ± 144	407.2 ± 141.6	357.7 ± 136.2
Alcohol intake (g/d)	6.5 ± 10.2	6.6 ± 9.2	7.2 ± 10.6	7.1 ± 11.2	6.2 ± 9.5
Fiber intake (g/d)	23.1 ± 8.4	25.1 ± 9	26.4 ± 9	27.8 ± 10.1	30.1 ± 11.9
Micronutrients					
Fe (mg/d)	16.3 ± 5	16.7 ± 4.9	17 ± 5.1	17.2 ± 5.3	16.9 ± 5.4
Cr (µg/d)	88.9 ± 34.3	86.3 ± 31.6	83.7 ± 31.7	81 ± 31.4	76.5 ± 30.7
I (mg/d)	332.3 ± 166.8	310.7 ± 156.1	290.9 ± 151.3	284.5 ± 152.2	300.2 ± 165.5
K (mg/d)	4286 ± 1185	4481 ± 1287	4569 ± 1335	4655 ± 1392	4726 ± 1424
Mg (mg/d)	377.4 ± 94.1	393.4 ± 103	400.7 ± 109	409.1 ± 113.5	415.4 ± 118.3
Ca (mg/d)	1199 ± 398.1	1179 ± 383.6	1137 ± 379.9	1121 ± 379.2	1102 ± 371.9
P (mg/d)	1802 ± 429.7	1840 ± 443.1	1838 ± 456.8	1845 ± 474.3	1822 ± 473.8
Na (mg/d)	3442 ± 1711	3328 ± 1654	3125 ± 1567	2980 ± 1555	2565 ± 1369
Se (µg/d)	87.1 ± 30.4	92.9 ± 30.5	93.5 ± 30.3	94.4 ± 32.6	92.3 ± 33
Zn (mg/d)	14.3 ± 4.8	15 ± 5.6	15.7 ± 6.4	16.7 ± 7.2	18.7 ± 8.8
Vitamin A (µg/d)	1612 ± 973.4	1788 ± 1068	1845 ± 1088	1942 ± 1142	2109 ± 1257
Vitamin B1 (mg/d)	1.8 ± 0.5	1.8 ± 0.5	1.8 ± 0.5	1.8 ± 0.5	1.7 ± 0.5
Vitamin B2 (mg/d)	2.1 ± 0.6	2.1 ± 0.6	2.1 ± 0.6	2.1 ± 0.6	2.1 ± 0.6
Vitamin B3 (mg/d)	38.9 ± 9.7	40.9 ± 10.3	41.7 ± 10.7	42 ± 11.1	40.4 ± 11.1
Vitamin B6 (mg/d)	2.3 ± 0.7	2.6 ± 0.7	2.7 ± 0.8	2.8 ± 0.8	2.9 ± 0.9
Vitamin B12 (mg/d)	7.9 ± 3.5	8.9 ± 3.9	9.5 ± 4.3	9.8 ± 4.4	9.5 ± 4.4
Vitamin C (mg/d)	229.1 ± 114.1	251.6 ± 120.2	265 ± 123	277.9 ± 130.9	300.7 ± 140.6
Vitamin D (µg /d)	4.1 ± 2.4	5.3 ± 3.2	6.2 ± 3.8	7.1 ± 4.2	7.6 ± 4.6
Vitamin E (mg/d)	6.2 ± 2.8	6.6 ± 2.8	6.7 ± 2.9	6.7 ± 2.9	6.6 ± 3.1
Folic acid (µg/d)	346.4 ± 131.1	376.1 ± 138.8	395.6 ± 145	414.5 ± 153.6	440 ± 162.1

^1^ Means ± SD are shown unless otherwise stated. Table adjusted for age and sex by the Inverse Probability Weighting method

^2^ Animal proteins comprised the following food groups: dairy products, eggs, meat, fish, processed and bakery products

^3^ Plant-based proteins comprised the following food groups: vegetables, fruits, nuts, pulses, cereals and grains, and drinks

ARG, arginine; HIS, histidine; ILE, isoleucine; LEU, leucine, LYS; lysine, MET, methionine; MUFAs, monounsaturated fatty acids; PHE, phenylalanine; PUFAs, polyunsaturated fatty acids; Q, quintile; TEI, total energy intake; THR, threonine; TRP, tryptophan; SFAs, saturated fatty acids; VAL, valine

**Fig. 2:**
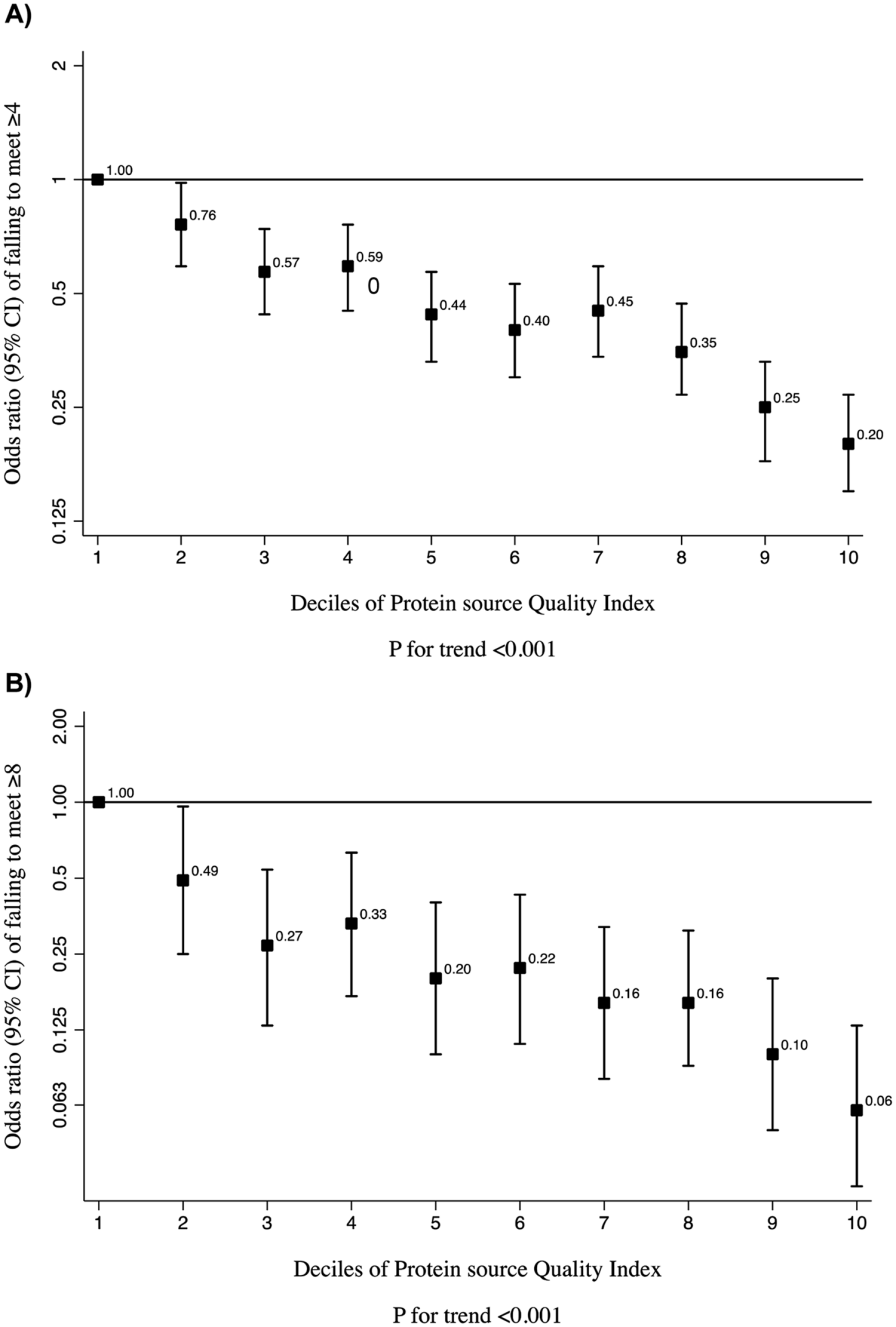
**A**, **B** OR and 95% confidence interval of unmet ≥ 4 or 8 Dietary Reference Intakes (DRI) respectively according to the deciles of protein source quality index in 17,535 participants of the SUN project (probabilistic approach). OR were adjusted for age (continuous), sex and education level (graduate, master, doctorate), energy intake (continuous), Mediterranean diet score (continuous), BMI (continuous), physical activity (metabolic equivalents-h/week), smoking status (no smoking, former, current < 15 cig/d, current ≥ 15 cig/d) and dietary supplement use (yes/no)

Table 3 presents the ORs of not meeting ≥ 4 or ≥ 8 DRI according to the quintiles of PQI. When we used the probabilistic approach method to predict the risk of failing to meet DRI, participants in the highest quintile of PQI had a 74% (OR = 0.26; IC 95% = 0.21, 0.32; *P* for trend < 0.001) and 89% (OR = 0.11; IC 95% = 0.06, 0.18; *P* for trend < 0.001) lower odds of failing to meet ≥ 4 and ≥ 8 DRI, respectively, compared to the first quintile in model 3. When we repeated the analyses for failing to meet ≥ 4 or ≥ 8 DRI using the EAR cut-off point approach, the results were very similar. Moreover, we repeated the analyses using deciles to calculate the PQI to investigate the risk of not to meet ≥ 4 DRI (Fig. 2A) or ≥ 8 DRI (Fig. 2B). Results from this analysis supported the robustness of our main findings across quintiles of PQI, with a significant *P* for trend in both cases.

**Table 3:** Odds ratio and 95% confident interval (95% CI) of failing to meet ≥ 4 and ≥ 8 Dietary Reference Intakes (DRI) according to the quintiles (Q) of PQI in 17,535 participants of the SUN Project

	Protein source Quality Index					P for trend
	Q1 (lowest quality)	Q2	Q3	Q4	Q5 (highest quality)	
*N*	3507	3507	3507	3507	3507	
*Probabilistic approach*						
Failing to meet ≥ 4 DRI (%)	13.1	11.3	10.8	11.3	10.8	
Crude	1 (Ref.)	0.85 (0.73–0.97)	0.79 (0.69–0.91)	0.81 (0.70–0.93)	0.75 (0.65–0.87)	< 0.001
Model 1	1 (Ref.)	0.86 (0.75–0.99)	0.83 (0.72–0.96)	0.85 (0.74–0.99)	0.84 (0.72–0.97)	0.028
Model 2	1 (Ref.)	0.57 (0.47–0.68)	0.37 (0.30–0.44)	0.29 (0.24–0.35)	0.13 (0.10–0.15)	< 0.001
Model 3	1 (Ref.)	0.66 (0.55–0.80)	0.48 (0.40–0.59)	0.46 (0.38–0.57)	0.26 (0.21–0.32)	< 0.001
Failing to meet ≥ 8 DRI (%)	2	1.3	1.5	1.6	1.9	
Crude	1 (Ref.)	0.68 (0.47–1.00)	0.76 (0.52–1.09)	0.83 (0.58–1.19)	0.94 (0.66–1.33)	0.932
Model 1	1 (Ref.)	0.69 (0.47–1.00)	0.77 (0.53–1.11)	0.84 (0.58–1.20)	0.97 (0.67–1.39)	0.843
Model 2	1 (Ref.)	0.40 (0.25–0.66)	0.27 (0.17–0.44)	0.21 (0.13–0.33)	0.09 (0.06–0.15)	< 0.001
Model 3	1 (Ref.)	0.41 (0.25–0.67)	0.29 (0.18–0.48)	0.22 (0.14–0.37)	0.11 (0.06–0.18)	< 0.001
*Cut-point approach*						
Failing to meet ≥ 4 DRI (%)	29	24.3	23.8	21.9	21.1	
Crude	1 (Ref.)	0.76 (0.68–0.84)	0.71 (0.63–0.78)	0.61 (0.54–0.68)	0.55 (0.49–0.62)	< 0.001
Model 1	1 (Ref.)	0.78 (0.70–0.86)	0.76 (0.68–0.84)	0.67 (0.60–0.74)	0.66 (0.59–0.74)	< 0.001
Model 2	1 (Ref.)	0.51 (0.44–0.58)	0.35 (0.31–0.41)	0.22 (0.19–0.26)	0.11 (0.09–0.13)	< 0.001
Model 3	1 (Ref.)	0.63 (0.54–0.72)	0.53 (0.46–0.61)	0.41 (0.35–0.48)	0.27 (0.23–0.32)	< 0.001
Failing to meet ≥ 8 DRI (%)	5.7	4	4.4	4.6	4.8	
Crude	1 (Ref.)	0.72 (0.58–0.90)	0.78 (0.63–0.96)	0.78 (0.63–0.97)	0.79 (0.64–0.98)	0.089
Model 1	1 (Ref.)	0.74 (0.59–0.92)	0.82 (0.66–1.02)	0.83 (0.67–1.04)	0.90 (0.72–1.13)	0.600
Model 2	1 (Ref.)	0.43 (0.32–0.57)	0.31 (0.24–0.42)	0.23 (0.17–0.31)	0.10 (0.07–0.14)	< 0.001
Model 3	1 (Ref.)	0.48 (0.36–0.64)	0.40 (0.29–0.53)	0.34 (0.25–0.46)	0.19 (0.13–0.26)	< 0.001

Model 1 adjusted for age (continuous), sex and education level (graduate, master, and doctorate)

Model 2 additionally adjusted for energy intake (continuous)

Model 3 additionally adjusted for the Mediterranean diet score (continuous), BMI (continuous), physical activity (METs-h/week), smoking status (no smoking, former, current < 15 cig/d, and current ≥ 15 cig/d) and dietary supplement use (yes/no)

When we analyzed the prevalence of participants with unmet EAR for each micronutrient (Supplemental Table 4), we found higher percentages among participants in the lowest quintile of PQI.

**Fig. 3:**
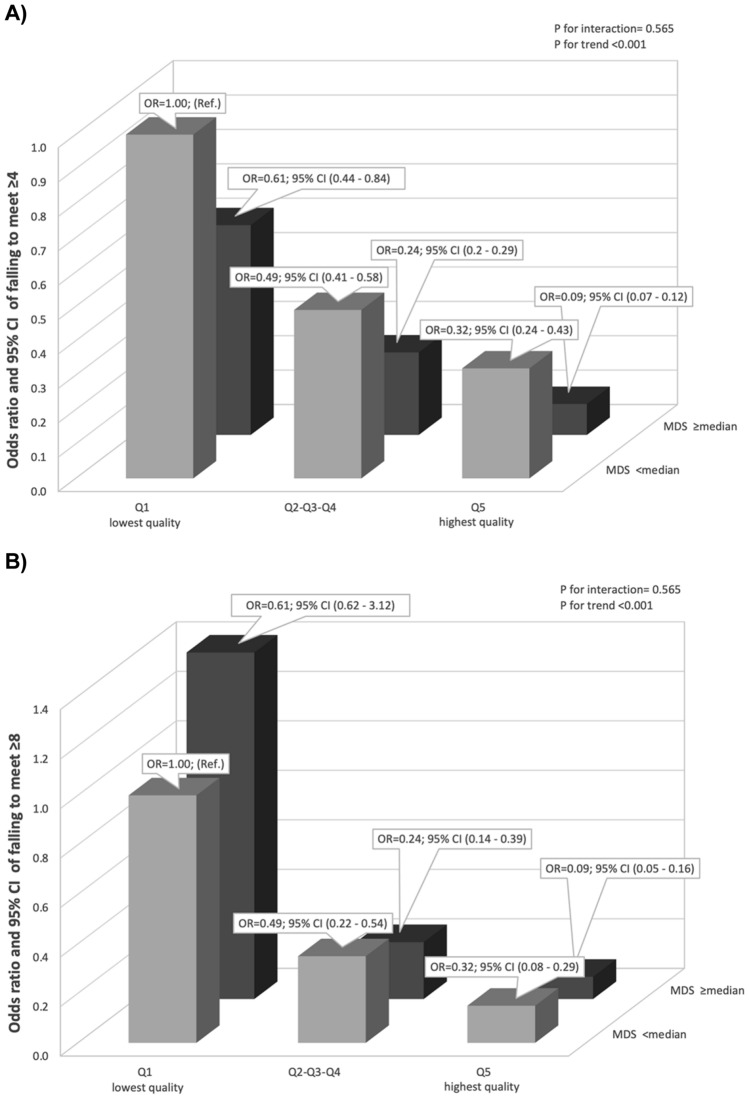
**A**, **B** OR and 95% CI of unmet ≥ 4 or 8 Dietary Reference Intakes respectively according to PQI and adherence to Mediterranean diet (MedDiet) in 17,535 participants of the SUN Project. OR were adjusted for age (continuous), sex and education level (graduate, master, doctorate), energy intake (continuous), BMI (continuous), physical activity (MET-h/week), smoking status (no smoking, former, current < 15 cig/d, current ≥ 15 cig/d) and dietary supplement use (yes/no). Abbreviations: Q, Quintiles, MDS, Mediterranean Diet

Figure 3A, B shows the OR for unmet EAR for failing to meet ≥ 4 or ≥ 8 DRI according to the joint classification by PQI and MDS. No significant interaction was observed (*P* for interaction = 0.565) although participants with a higher PQI and higher adherence to the MedDiet presented a lower risk of unmet EAR compared to the lowest category of PQI and MedDiet. We also assessed the prevalence of inadequate micronutrient intake according to quintiles of PQI and stratified by level of adherence to the MedDiet. We observed that participants with higher adherence to MDS vs. lower adherence showed lower prevalence of inadequate Fe, Cr, K, Mg, Se and all vitamins (A, B complex, C, D and E) (*P* between groups < 0.001 in all except for B3 with *P* = 0.179 and B12 with *P* = 0.286), and statistically significant higher prevalence of inadequate, Ca and Zn in participants with higher vs. lower PQI score (Supplemental Tables 5 and 6).

Pearson’s correlations showed that total protein intake was strongly associated with animal protein because there is a high dietary content of animal food sources compared to plant protein (Supplemental Figure 1). A strong association of total protein was found with intakes of Mg, P, vitamins B1, B2, B3, and B6, present mainly in foods of animal origin, and a moderate association with intakes of vegetable protein, I, Zn, Na, K, Cr, folic acid and vitamin B12, and vitamin E in our cohort (Supplemental Figure 1). Intake of animal protein was moderately associated with all minerals and vitamin B1, B6 and B12, and strongly associated with vitamin B2 and B3 (found mainly in sources of animal origin). On the contrary, intake of vegetable protein was strongly associated with intakes of Fe, Mg, K, and vitamin B1, and moderately associated with the rest of the minerals except I and Na (Supplemental Figure 1).

Finally, we performed 4 sensitivity analyses: (I) adjusting for total energy intake using the residual method; (II) redefining the PQI using the HPPQI; (III) no-excluding participants with implausible intakes; and (IV) applying different exclusions energy intakes criteria according to percentiles (< P5 and > P95; < P1 and > P99). When we adjusted the score using the residual method, the results showed that a highest PQI had a 82% (OR = 0.18; IC 95% = 0.14, 0.24; *P* for trend < 0.001) and 97% (OR = 0.03; IC 95% = 0.01, 0.10; *P* for trend < 0.001) lower risk of falling to meet ≥ 4 or ≥ 8 DRI, respectively, as compared to the lowest quintile in the probabilistic approach model. When we used the cut-off approach model using the residual method results were apparently similar (OR = 0.22; IC 95% = 0.18, 0.26; *P* for trend < 0.001; and OR = 0.08; IC 95% = 0.05, 0.14; *P* for trend < 0.001, of falling to meet ≥ 4 or ≥ 8 DRI respectively). When we used the HPPQI, the odds of participants in the highest quintile of HPPQI of not meeting ≥ 4 or ≥ 8 DRI was lower than participants in the lowest HPPQI. The OR of unmet ≥ 4 DRI was 0.50 (IC 95% = 0.43, 0.59; *P* for trend < 0.001) for the cut-off points and 0.60 (IC 95% = 0.49, 0.73; *P* for trend < 0.001) for the probability approach. The OR of unmet ≥ 8 DRI was 0.55 (IC 95% = 0.41, 0.75; *P* for trend < 0.001) for the cut-off points approach and 0.49 (IC 95% = 0.30, 0.78; *P* for trend = 0.004) for the probabilistic approach. These results show that the odds of unmet DRI between extreme quintiles were lower when the quality of protein intake was measured with the dietary PQI than with the HPPQI. Lastly, after no-excluding participants with implausible energy intakes and after applying different exclusions energy intakes criteria according to percentiles (P95;  P99), the results were very similar to our main analysis (Supplemental Figure 2).

## Discussion

To our knowledge, the present study is the first to develop a score of a dietary PQI according to the content of essential amino acids, the health benefits and environmental impact of the protein sources, and to investigate the association between the PQI and the adequacy of micronutrient intake in a large Mediterranean cohort. Participants with higher PQI scores (PQI range in Q5 1.3 to 41.1) had lower energy intake with a higher consumption of protein groups with better quality (fish, white meat, legumes, eggs, nuts, low-fat dairy products, and whole grains) compared to those with a lower reference score (PQI range in Q1 0 to < 0.5). In addition, they had better lifestyles (more active, less snacking between meals, more special diets, and greater adherence to the MedDiet). Since total protein intake is relatively stable [46], the PQI likely promotes a higher intake of healthy proteins at the expense of reducing the intake of unhealthy proteins. We found that participants with higher PQI had a lower risk of having an inadequate intake of micronutrients. We found that the odds of not meeting ≥ 4 and ≥ 8 DRIs were lower when we controlled in multivariable model 2 for energy intake. These results suggest that total energy intake was an important confounding factor. When we adjusted in model 3 for different lifestyles, the odds of not meeting ≥ 4 and ≥ 8 DRIs were also lower, although less pronounced. In addition, those participants with better adherence to the MedDiet were more likely to meet micronutrient recommendations compared to those with lowest adherence. No significant interaction was observed between the PQI and the level of adherence to the MDS.

Few studies previously addressed the association between protein quantity with diet quality and micronutrient adequacy on adult population. In a cross-sectional study in the United States high protein density was associated with a greater probability of nutritional adequacy, independently of the intake of fruits and vegetables [19]. Findings from the French Individual and National Consumption Survey 2 suggested that plant protein is a robust marker of a healthy and quality diet, whereas total animal protein includes different subtypes of animal protein that largely vary in their relationship with diet quality [20]. Lastly, they conclude that plant-based protein intake is a general and robust marker of nutrient adequacy of the diet, and hence of a healthy diet. In other studies, Canadian and American adults who reported a higher consumption of proteins from plant-based foods has reported an enhanced micronutrient intake resulting in lower individuals who were below the EAR for vitamins B1, B6, and folic acid, Fe, Mg, P, and Zn compared with non-consumers [47, 48].

Dietary protein patterns constitute strong elements in the background structure of the dietary intake of a general population and are associated with different nutrient profiles [18]. In our study, a higher PQI is associated with higher protein consumption, but specifically it reflects a higher intake of healthy plant and animal protein sources with lower environmental impact (such as fish, lean meat, and reduced-fat dairy products) [49, 50]. One of the results of our study confirms that the relationship between animal protein and diet quality extends beyond the case of meat, and the intake of plant protein shows strong associations with patterns of micronutrient intake (Supplemental Figure 1). For this reason, a combined consumption of protein of animal and vegetable origin would be able to fill possible micronutrient lacks in the diet and it will ensure to achieve the necessary micronutrient profile. Our results suggest that multidimensional assessment of protein source quality also appears to be important in adequate micronutrient intake, and an approach based solely on protein quantity could limit health benefits and favor a higher environmental footprint. Thus, in agreement with other recent dietary quality indexes that have assessed the carbohydrate and fat quality [21], the quality of protein sources is strongly considered as a link to the micronutrient adequacy of the diet. Moreover, our results also justify the relevance of using a global assessment tool of nutrient adequacy when studying the relationship between the intake of animal- and vegetable-source proteins and the quality of the micronutrient contribution or other variables related to health. It should be noted that good quality of protein intake does not require the complete elimination of animal products.

Adequate dietary protein intake provides a source of micronutrients as well as amino acids, including the 9 essential amino acids. The dietary PQI was calculated according to the content of essential amino acids in each food group. Animal-source foods, such as meats, dairy products, eggs, fish or seafood, contain higher amounts of amino acids than plant-source foods, such as cereals, vegetables, potatoes, legumes, nuts, and seeds [8]. One of the reasons that defines the quality of protein in the diet refers to the concept of "limiting amino acid" [9]. The 2 amino acids most likely to be limiting are LYS and MET in pulses and cereals [9]. In this way, our index considers legumes and whole grains, advocating that both food groups can be part of a dietary pattern that, in addition to being healthy, provides an adequate number of micronutrients and in their proportion of quality amino acids. It is widely believed by the general population that many plant foods are completely lacking in specific amino acids, and therefore protein sufficiency cannot be supported by plant foods alone. However, all plant foods contain the 20 amino acids in the diet [9], as shown in Supplemental Figure 3. Thus, although the amount of essential amino acids is higher in foods of animal origin, the proportion of each amino acid essential in plant foods follows a similar distribution as in those of animal origin.

The MedDiet is an example that the combination of healthy animal-source proteins and vegetable-source proteins contribute to a sufficient amount of micronutrients in the diet, excellent sources of Zn, I, Se, Fe, Ca, K, P, Mg, Cr and vitamins B1, B2, B3, B6, B12, C, A, D and folic acid [19, 21, 51]. Several studies concluded that following a MedDiet pattern was associated with a lower risk of unmeet the EAR of micronutrients [21, 23]. In accordance with this, in our study those participants who adhered more to the MedDiet and scored a higher PQI would cover most of the micronutrients, but a possible inadequacy in I, Ca and Zn would have to be taken into account. To compensate the possible deficiency of these micronutrients, the substitution of meat for fish and seafood (main source of I); a higher consumption of small fish, such as sardines, anchovies, green leafy vegetables (secondary sources of Ca); and a higher consumption of nuts and legumes (secondary sources of Zn) could be recommended. Moreover, we found that regardless of the MedDiet, the effect of the PQI on the risk of nutritional inadequacy was not modified.

Our study has some limitations. First, to calculate the dietary PQI we did not take into account other food groups (fruits, vegetables, drinks, bakery, pastries and other processed products) associated with protein intake in the SUN cohort. However, the PQI includes those foods that contribute to the highest percentage of total protein intake (the 12 groups contribute 81.84% of total protein intake vs. an estimated mean of protein contribution of 86.84% according to the data available in the Spanish ANIBES study [52]). Similar percentage contribution was found in the National Health and Nutrition Examination Survey 2007–2010 [17] where the top food sources of protein achieved approximately 73% of total daily protein intake. Third, we used a self-reported FFQ to estimate food consumption and nutrient intake, which can be an important source of information bias. However, the FFQ is considered the most appropriate tool in epidemiology and the most practical and feasible tool to evaluate food habits in large epidemiological studies [53]. Besides, our sample comprised highly educated participants, so the data probably have fairly adequate quality [54], and the FFQ was previously validated in Spain and subsequently re-evaluated [24, 27, 28]. Fourth, there is currently a lack of scientific agreement on the health properties according to the type of dairy or red meat. Since the underpinning information in our study was collected from a FFQ, our score cannot disentangle any subtle differences between types of fat in dairy products (low-fat, semi-skim or whole dairy) and between red or processed meat. Fifth, total dietary intake of micronutrients could be underestimated, although we included the intake from foods and from dietary supplements in the data analysis (without considering the intake of fortified foods or medication that participants might be consuming). Sixth, our study assessed the probability of adequacy but did not indicate nutrient deficiencies which should be confirmed by biomarkers of nutrient intake. Seventh, the dietary PQI definition took into account the environmental effect according to the Dietary Guidelines for Americans (2020–2025) and FAO, but it did not assess the life cycle assessment that is a common choice for assessment the environmental impact of food measure as the resource use, pollutant emissions, and other potential impacts of foods [36]. Eighth, we did not collect information on the socioeconomic status of participants. Since all participants in the SUN cohort are university graduates, they are not very heterogeneous regarding their socioeconomic and educational status. However, we are not able to know whether lack of affordability of high-quality foods can be related to suboptimal intake of some micronutrients and to the overall health-related lifestyles in participants of our cohort. Ninth, we used international sources, such as the USDA and German food databases, to obtain information on essential amino acids, which may not ideally reflect the content of essential amino acids in our Spanish population.

The strengths of the present study are that all data were obtained from a Mediterranean cohort with a relatively large sample size and a high response rate (91%). Second, the components of dietary PQI were based on international evidence and recommendations. Third, our analysis was obtained using two different methods to estimate nutrient intake adequacy: the probabilistic approach and the EAR cut-point approach. Fourth, the FFQ was validated in Spain and subsequently re-evaluated [24, 27, 28].

In conclusion, it is possible to develop a quality indicator of protein intake considering not only quantity and amino acid content of proteins but also the health and environmental effects of each protein food source. A higher protein source quality is associated with better micronutrient intake adequacy in this long Mediterranean cohort. These results could contribute to the development of future dietary recommendations regarding the quantity and quality of protein intake. It would be interesting to incorporate the impact on human and environmental health into future international recommendations on protein intake. Protein consumption should be promoted, in moderate amounts and within the framework of a balanced diet the healthiest and sustainable foods. In addition, we believe that investigating this multidimensional concept of dietary protein quality should be transferred to other populations to analyze how it would influence nitrogen balance or body mass/protein balance using the PQI. Therefore, more studies are required in other populations to evaluate the efficacy and validity of this new dietary protein quality index. Further studies are required in other populations to evaluate the efficacy of this novel dietary quality index.

## Supplementary Information

Below is the link to the electronic supplementary material.

Supplementary file1 (DOCX 20275 KB)

## Data Availability

This study uses data from the *Seguimiento Universidad de Navarra* (SUN) cohort. All data and materials as well as software application or custom code used during the current study shall be made available from the corresponding author on reasonable request.

## Abbreviations

AI: Adequate intake
DRI: Dietary References intakes
EAR: Estimated Average Requirement
FFQ: Food Frequency Questionnaire
HPPQI: Harvard's healthy eating Plate Protein source Quality Index
His: Histidine
Ile: Isoleucine
Leu: Leucine
Lys: Lysine
Met: Methionine
MDS: Mediterranean Diet Score
MEDAS: Mediterranean Diet Adherence Screener
OR: Odds Ratios
Phe: Phenylalanine
PQI: Protein source Quality Index
PREDIMED: PREvención con DIeta MEDiterránea
RDA: Recommended Dietary Allowance
SUN: Seguimiento Universidad de Navarra
Thr: Threonine
Trp: Tryptophan
UL: Tolerable upper level values
Val: Valine
