# Supplementary material for: Association between a new dietary protein quality index and micronutrient intake adequacy: a cross-sectional study in a young adult Spanish Mediterranean cohort
Source: Eur J Nutr. 2022 Sep 10;62(1):419–32. doi: 10.1007/s00394-022-02991-z (PMC9899725; doi:10.1007/s00394-022-02991-z)
Supplement: Supplementary file 1 — Supplementary file1 (DOCX 20275 KB) [file 394_2022_2991_MOESM1_ESM.docx]

**Supplemental table 1**. Protein contribution (%) of food groups according to the total energy intake of proteins in participants of the SUN Project (N = 22,864).

| **Food group** | **Percentage** | **SD** | **Mean ^a^ (%)** | **Spanish ANIBES Study ^1^** |
| --- | --- | --- | --- | --- |
| **Red meats** | 22.95 | 9.40 | 22.95 | 16.57 ^b^ |
| **Full-fat dairy products** | 12.48 | 8.65 | 12.48 | 8.85 ^c^ |
| **Potatoes and refined grains** | 9.62 | 5.93 | 9.62 | 8.69 ^d^ |
| **Lean meats** | 9.05 | 6.10 | 9.05 | 16.57 ^b^ |
| Vegetables | 7.30 | 4.44 |  | 3.79 |
| **Lean fish** | 5.77 | 3.86 | 5.77 | 3.54 ^e^ |
| **Reduced-fat dairy products** | 5.43 | 7.29 | 5.43 | 8.85 ^c^ |
| **Pulses** | 4.65 | 3.64 | 4.65 | 3.32 |
| **Fat fish** | 4.40 | 3.67 | 4.40 | 3.54 ^e^ |
| Processed products | 3.90 | 3.50 |  | - |
| Pastries | 3.60 | 4.37 |  | 2.77 |
| **Eggs** | 2.76 | 1.91 | 2.76 | 4.68 |
| Fruit | 2.59 | 2.23 |  | 1.90 |
| **Seafood** | 2.49 | 2.26 | 2.49 | 3.54 ^e^ |
| **Nuts** | 1.19 | 2.07 | 1.19 | - |
| **Whole grains** | 1.05 | 2.48 | 1.05 | 8.69 ^d^ |
| Drinks | 0.77 | 0.66 |  | - |
| **Total protein contribution** | **100%** |  | **81.84%** | **86.84% ^f^** |
|  |  |  |  |  |

^a^ Mean of protein contribution of PQI components. The 12 food groups of interest are shown in bold.

^b^ It is shown a mean data (divided in 2) of meat and meat products in the Spanish ANIBES study (the total value is 33.14%).

^c^ It is shown a mean data (divided in 2) of milk and dairy products in the Spanish ANIBES study (the total value is 17.17%).

^d^ It is shown a mean data (divided in 2) of cereals and grains products in the Spanish ANIBES study (the total value is 17.38%).

^e^ It is shown a mean data (divided in 3) of fish and seafood products in the Spanish ANIBES study (the total value is 10.63%).

^f^ Estimated mean of protein contribution of PQI components according with the data available in in the Spanish ANIBES study.

REFERENCES

1. Ruiz, E., Ávila, J. M., Valero, T., Del Pozo, S., Rodriguez, P., Aranceta-Bartrina, J., Gil, Á., González-Gross, M., Ortega, R. M., Serra-Majem, L., & Varela-Moreiras, G. (2016). Macronutrient Distribution and Dietary Sources in the Spanish Population: Findings from the ANIBES Study. Nutrients, 8(3), 177. https://doi.org/10.3390/nu8030177

**Supplemental table 2**. Content of essential amino acid (mg) according to food groups per 100g (2,3).

|  | **Essential amino acid content per 100g** | | | | | | | | |  | | |
| --- | --- | --- | --- | --- | --- | --- | --- | --- | --- | --- | --- | --- |
|  | **HIS** | **ILE** | **LEU** | **LYS** | **MET** | **PHE** | **THR** | **TRP** | **VAL** | | **Total AA content** | **Sequence*** |
| **Food group** | **(mg)** | **(mg)** | **(mg)** | **(mg)** | **(mg)** | **(mg)** | **(mg)** | **(mg)** | **(mg)** | | **(mg)** |  |
| **Fat fish** | 669.1 | 1078.2 | 1736.4 | 1846.4 | 626.4 | 848 | 1011.8 | 224.6 | 1246.4 | | 9287.3 | 1 |
| **Pulses** | 617.8 | 1213.3 | 1846.3 | 1662.5 | 248.8 | 1158.8 | 984.6 | 216.8 | 1255.8 | | 9204.7 | 2 |
| **Lean fish** | 458.9 | 1011.7 | 1557.3 | 2195.3 | 675.6 | 752.3 | 931 | 273.9 | 981 | | 8837 | 3 |
| **Lean meat** | 543.3 | 1133.3 | 1645.6 | 1894.4 | 577.8 | 836.7 | 955.6 | 140 | 1060 | | 8786.7 | 4 |
| **Seafood** | 449.6 | 946.9 | 1568.1 | 1723.1 | 628.5 | 808.1 | 930.8 | 209.1 | 1006.1 | | 8270.3 | 5 |
| **Red meat** | 627.1 | 886.4 | 1529.8 | 1645 | 463.1 | 784.3 | 863.8 | 218 | 1071.5 | | 8089 | 6 |
| **Eggs** | 330 | 930 | 1260 | 890 | 450 | 800 | 710 | 230 | 1120 | | 6720 | 7 |
| **Nuts** | 435.7 | 990 | 1498.3 | 642.9 | 231.4 | 917.1 | 584.3 | 237.1 | 1050 | | 6586.8 | 8 |
| **Potatoes and refined grains** | 440 | 655 | 1302.5 | 779.2 | 193.3 | 769.2 | 595.8 | 150.8 | 834.2 | | 5720 | 9 |
| **Full-fat dairy products** | 228.2 | 430.3 | 770.1 | 653 | 209.5 | 396 | 324.5 | 101.6 | 518.7 | | 3631.9 | 10 |
| **Reduced-fat dairy products** | 231.3 | 427 | 714.3 | 630.7 | 199.7 | 358 | 317.7 | 81 | 460.7 | | 3420.4 | 11 |
| **Whole grains** | 200 | 332.5 | 565 | 255 | 87.5 | 440 | 250 | 67.5 | 372.5 | | 2570 | 12 |
| **Pastries** | 125.8 | 249.3 | 435.5 | 266.8 | 111.2 | 277 | 210.7 | 75.2 | 291 | | 2042.5 | 13 |
| **Processed products** | 71.2 | 122.9 | 217.8 | 161.1 | 51.3 | 124 | 101.8 | 37.4 | 146.2 | | 1033.7 | 14 |
| **Vegetables** | 31.2 | 70.4 | 93.5 | 83.2 | 19.2 | 60.1 | 62.3 | 21 | 79.3 | | 520.2 | 15 |
| **Fruits** | 21 | 28.7 | 45.4 | 44.9 | 12.2 | 29.9 | 30.8 | 10.3 | 38 | | 261.2 | 16 |
| **Drinks** | 0.8 | 1.8 | 2.9 | 2.5 | 0.7 | 2 | 1.8 | 0.6 | 2.5 | | 15.6 | 17 |

Abbreviations: histidine (HIS), isoleucine, (ILE) leucine (LEU), lysine (LYS), methionine (MET), phenylalanine (PHE), threonine, (THR) tryptophan (TRP), and valine (VAL). The heat map color scale was established individually for each amino acid and nutrient. The colors refer to the essential amino acid content taken from the food composition tables. Red color means lower essential amino acid content vs. green color means higher essential amino acid content.

*Position based on essential amino acid content by food group. E.g., 1 means higher total essential amino acid content and 17 means lower essential total amino acid content.

REFERENCES

2. U.S. DEPARTMENT OF AGRICULTURE. Food data [Internet]. [cited 2021 May 4]. Available from: <https://fdc.nal.usda.gov>

3. Siegfried W. Souci, W Fachmann, Heinrich Kraut. Food Composition and Nutrition Tables, 7th revised and completed edition. 2008.

**Supplemental table 3**. Summary of factors that was considered to the creation of PQI.

|  | **Factors to consider** | | | |  |
| --- | --- | --- | --- | --- | --- |
| **Food group** | **Protein content** | **Essential amino acids** | **Effects on health** | **CO_2_e per kg edible weight ^1^** | Points |
| **Fat fish** | + | + | + | + | 4 |
| **Lean fish** | + | + | + | + | 4 |
| **Seafood** | + | + | + | + | 4 |
| **Lean meat** | + | + | + | + | 4 |
| **Red meat** | + | + | – | – | 2 |
| **Pulses** | + | + | + | + | 4 |
| **Eggs** | + | + | + | + | 4 |
| **Nuts** | + | + | + | + | 4 |
| **Potatoes and refined grains** | + | + | – | + | 3 |
| **Full-fat dairy products** | + | + | – | + | 3 |
| **Reduced-fat dairy products** | + | + | + | + | 4 |
| **Whole grains** | + | + | + | + | 4 |

^1^ Developments in national healthy and sustainable dietary guidelines: a state of play assessment (FAO, 2016). Low (<1 kg CO_2_ equivalent per kg edible weight) and medium (1-4 kg CO_2_ equivalent per kg edible weight) climate impact from primary production of food are expressed with a “+”, while high (>4 kg CO_2_ equivalent per kg edible weight) climate impact from primary production of food are expressed with a “–“.

REFERENCES:

4. 2020-2025 Dietary Guidelines for Americans. U. S. Department of Agriculture and Department of Health and Human Services. https://www.dietaryguidelines.gov/sites/default/files/2020-12/Dietary_Guidelines_for_Americans_2020-2025.pdf [Internet]. Available from: https://www.dietaryguidelines.gov/sites/default/files/2020-12/Dietary_Guidelines_for_Americans_2020-2025.pdf

5. Gonzalez Fischer C, Garnett T. Developments in national healthy and sustainable dietary guidelines: a state of play assessment (FAO). 2016. 1–80 p.

**Supplemental table 4**. Prevalence of inadequate micronutrient intake of participants in the SUN cohort with intakes below Estimated Average Requirement (EAR) according to quintiles of the Protein Quality Index* (N=17,535).

|  | **Protein source Quality Index** | | | | |  |
| --- | --- | --- | --- | --- | --- | --- |
| **Micronutrients** | **Q1 (lowest quality)** | **Q2** | **Q3** | **Q4** | **Q5 (higher quality)** | **P for trend** |
|  |  |  |  |  |  |  |
| N | 3,507 | 3,507 | 3,507 | 3,507 | 3,507 |  |
| N micronutrients with intakes not meeting the EAR (mean and SD) | 3.9 (2.5) | 3.4 (2.5) | 2.9 (2.1) | 2.4 (1.8) | 1.9 (1.3) | <0.001 |
|  |  |  |  |  |  |  |
| Fe | 1.9 | 1.8 | 1 | 0.8 | 0.3 | <0.001 |
| Cr | 2.6 | 2 | 1.5 | 0.9 | 0.3 | <0.001 |
| I | 9.1 | 10.5 | 9.3 | 8.5 | 6.2 | <0.001 |
| K | 22.7 | 16.8 | 10.5 | 6.4 | 1.8 | <0.001 |
| Mg | 34 | 26.7 | 18.8 | 11.7 | 3.7 | <0.001 |
| Ca | 20.5 | 22 | 20.7 | 17.6 | 11.2 | <0.001 |
| P | 0.4 | 0.3 | 0.1 | 0.1 | 0 | <0.001 |
| Se | 8 | 5.6 | 4.2 | 3.1 | 2.1 | <0.001 |
| Zn | 10.2 | 9.2 | 6.1 | 3.7 | 1.7 | <0.001 |
| Vitamin A | 11.5 | 7.3 | 4.6 | 3.8 | 1.9 | <0.001 |
| Vitamin B1 | 6.2 | 5.4 | 3.7 | 2.4 | 0.8 | <0.001 |
| Vitamin B2 | 3.6 | 2.9 | 1.9 | 1.2 | 0.2 | <0.001 |
| Vitamin B3 | 0.4 | 0.3 | 0.1 | 0 | 0 | <0.001 |
| Vitamin B6 | 3.4 | 2.2 | 0.9 | 0.3 | 0.1 | <0.001 |
| Vitamin B12 | 1.4 | 1 | 0.5 | 0.6 | 0.7 | 0.001 |
| Vitamin C | 3.8 | 2.4 | 1.3 | 0.9 | 0.3 | <0.001 |
| Vitamin D | 95.1 | 86.3 | 74.4 | 62.7 | 51.6 | <0.001 |
| Vitamin E | 96.8 | 95.9 | 94.9 | 93.6 | 88.6 | <0.001 |
| Folic acid | 54.6 | 43.8 | 34.2 | 25.7 | 13.5 | <0.001 |
|  |  |  |  |  |  |  |

*We used the residual method adjustment (37) to create the PQI and nullify the correlation proteins that comprise the score and the energy intake.

Means and IC 95% are expressed in % of participants in the SUN cohort with intakes below Estimated Average Requirement (EAR) stratified by MedDiet adherence (<median and ≥median). Mediterranean diet adherence score range 0-9 points, <median range to 0-3 points and ≥median range to 4-9 points. P for trend was calculated assigning the median value to each quintile and considered the variable as continuous.

**Supplemental table 5**. Prevalence of inadequate minerals intake between extreme quintiles of PQI according to adherence of Mediterranean diet (<median and ≥median) (N=17,535).

|  | **Protein source Quality Index** | |  |  |  |  |
| --- | --- | --- | --- | --- | --- | --- |
| **Minerals** | **Q1 (lowest quality) n=3,507** | **Q5 (highest quality) n=3,507** | **Diff. PQI quintiles** | **P for trend** | **P value between extreme quintiles** | **P value between groups of MDS** |
| N in MDS <median | 2,603/3,507 | 542/3,507 |  |  |  |  |
| N in MDS ≥median | 904/3,507 | 2,965/3,507 |  |  |  |  |
|  | **Means in % (95% CI)** | **Means in % (95% CI)** | **Q5 vs. Q1** |  |  |  |
|  |  |  |  |  |  |  |
| **Fe** |  |  |  |  |  |  |
| *MDS <median* | 2.5 (1.9 to 3.1) | 1.1 (0.2 to 2.0) | –1.4 | 0.036 | 0.047 |  |
| *MDS ≥median* | 0.1 (–0.1 to 0.3) | 0.1 (0 to 0.2) | 0 | 0.010 | 0.938 |  |
| *Diff. in MDS* | –2.4 | –1.1 |  |  |  | <0.001 |
|  |  |  |  |  |  |  |
| **Cr** |  |  |  |  |  |  |
| *MDS <median* | 2.9 (2.3 to 3.6) | 1.1 (0.2 to 2.0) | –1.8 | 0.001 | 0.016 |  |
| *MDS ≥median* | 1.5 (0.7 to 2.4) | 0.2 (0 to 0.4) | –1.3 | <0.001 | <0.001 |  |
| *Diff. in MDS* | –1.4 | –0.9 |  |  |  | <0.001 |
|  |  |  |  |  |  |  |
| **I** |  |  |  |  |  |  |
| *MDS <median* | 7.9 (6.9 to 9.0) | 5.5 (3.6 to 7.5) | –2.4 | 0.309 | 0.056 |  |
| *MDS ≥median* | 12.4 (10.2 to 14.5) | 6.3 (5.4 to 7.2) | –6.1 | <0.001 | <0.001 |  |
| *Diff. in MDS* | +4.5 | +0.8 |  |  |  | 0.176 |
|  |  |  |  |  |  |  |
| **K** |  |  |  |  |  |  |
| *MDS <median* | 25.7 (24.0 to 27.4) | 3.7 (2.1 to 5.3) | –22 | <0.001 | <0.001 |  |
| *MDS ≥median* | 13.9 (11.7 to 16.2) | 1.5 (1.0 to 1.9) | –12.4 | <0.001 | <0.001 |  |
| *Diff. in MDS* | –11.8 | –2.2 |  |  |  | <0.001 |
|  |  |  |  |  |  |  |
| **Mg** |  |  |  |  |  |  |
| *MDS <median* | 36.1 (34.2 to 37.9) | 6.1 (4.1 to 8.1) | –30 | <0.001 | <0.001 |  |
| *MDS ≥median* | 27.9 (24.9 to 30.8) | 3.3 (2.7 to 3.9) | –24.6 | <0.001 | <0.001 |  |
| *Diff. in MDS* | –8.2 | –2.8 |  |  |  | <0.001 |
|  |  |  |  |  |  |  |
| **Ca** |  |  |  |  |  |  |
| *MDS <median* | 20.0 (18.5 to 21.6) | 11.1 (8.4 to 13.7) | –8.9 | <0.001 | <0.001 |  |
| *MDS ≥median* | 21.9 (19.2 to 24.6) | 11.3 (10.1 to 12.4) | –10.6 | <0.001 | <0.001 |  |
| *Diff. in MDS* | +1.9 | +0.2 |  |  |  | <0.001 |
|  |  |  |  |  |  |  |
| **P** |  |  |  |  |  |  |
| *MDS <median* | 0.4 (0.2 to 0.7) | 0 | –0.4 | 0.044 | 0.130 |  |
| *MDS ≥median* | 0.4 (0 to 0.9) | 0 | –0.4 | <0.001 | <0.001 |  |
| *Diff. in MDS* | 0 | 0 |  |  |  | 0.003 |
|  |  |  |  |  |  |  |
| **Se** |  |  |  |  |  |  |
| *MDS <median* | 8.3 (7.2 to 9.4) | 3.5 (2.0 to 5.1) | –4.8 | <0.001 | <0.001 |  |
| *MDS ≥median* | 7.2 (5.5 to 8.9) | 1.9 (1.4 to 2.4) | –5.3 | <0.001 | <0.001 |  |
| *Diff. in MDS* | –1.1 | –2.6 |  |  |  | <0.001 |
|  |  |  |  |  |  |  |
| **Zn** |  |  |  |  |  |  |
| *MDS <median* | 10.4 (9.3 to 11.6) | 1.1 (0.2 to 2.0) | –9.3 | <0.001 | <0.001 |  |
| *MDS ≥median* | 9.4 (7.5 to 11.3) | 1.9 (1.4 to 2.3) | –7.5 | <0.001 | <0.001 |  |
| *Diff. in MDS* | –1 | +0.8 |  |  |  | <0.001 |
|  |  |  |  |  |  |  |

Means and IC 95% are expressed in % of participants in the SUN cohort with intakes below Estimated Average Requirement (EAR) stratified by MDS adherence (<median and ≥median). Mediterranean diet (MDS) adherence score range 0-9 points, <median range to 0-3 points and ≥median range to 4-9 points. P for trend was calculated assigning the median value to each quintile and considered the variable as continuous.

**Supplemental table 6**. Prevalence of inadequate vitamins intake between extreme quintiles of PQI according to adherence of Mediterranean diet (<median and ≥median) (N=17,535).

|  | **Protein source Quality Index** | |  |  |  |  |
| --- | --- | --- | --- | --- | --- | --- |
| **Vitamins** | **Q1 (lowest quality) n=3,507** | **Q5 (highest quality) n=3,507** | **Diff. PQI quintiles** | **P for trend** | **P value between extreme quintiles** | **P value between groups of MDS** |
| N in MDS <median | 2,603/3,507 | 542/3,507 |  |  |  |  |
| N in MDS ≥median | 904/3,507 | 2,965/3,507 |  |  |  |  |
|  | **Means in % (95% CI)** | **Means in % (95% CI)** | **Q5 vs. Q1** |  |  |  |
|  |  |  |  |  |  |  |
| **Vitamin A** |  |  |  |  |  |  |
| *MDS <median* | 13.1 (11.8 to 14.4) | 4.4 (2.7 to 6.2) | –8.7 | <0.001 | <0.001 |  |
| *MDS ≥median* | 7.0 (5.3 to 8.6) | 1.4 (1.0 to 1.8) | –5.6 | <0.001 | <0.001 |  |
| *Diff. in MDS* | –6.1 | –3 |  |  |  | <0.001 |
|  |  |  |  |  |  |  |
| **Vitamin B1** |  |  |  |  |  |  |
| *MDS <median* | 7.2 (6.2 to 8.2) | 2.2 (1.0 to 3.5) | –5 | <0.001 | <0.001 |  |
| *MDS ≥median* | 3.5 (2.3 to 4.7) | 0.5 (0.3 to 0.8) | –3 | <0.001 | <0.001 |  |
| *Diff. in MDS* | –3.7 | ­–1.7 |  |  |  | <0.001 |
|  |  |  |  |  |  |  |
| **Vitamin B2** |  |  |  |  |  |  |
| *MDS <median* | 3.5 (2.8 to 4.2) | 0.2 (–0.2 to 0.5) | –3.3 | <0.001 | <0.001 |  |
| *MDS ≥median* | 3.7 (2.4 to 4.9) | 0.2 (0.1 to 0.4) | –3.5 | <0.001 | <0.001 |  |
| *Diff. in MDS* | +0.2 | 0 |  |  |  | <0.001 |
|  |  |  |  |  |  |  |
| **Vitamin B3** |  |  |  |  |  |  |
| *MDS <median* | 0.3 (0.1 to 0.5) | 0 | –0.3 | 0.033 | 0.196 |  |
| *MDS ≥median* | 0.6 (0.1 to 1.0) | 0 | –0.6 | <0.001 | <0.001 |  |
| *Diff. in MDS* | +0.3 | 0 |  |  |  | 0.179 |
|  |  |  |  |  |  |  |
| **Vitamin B6** |  |  |  |  |  |  |
| *MDS <median* | 4.0 (3.2 to 4.7) | 0.7 (0 to 1.5) | –3.3 | <0.001 | <0.001 |  |
| *MDS ≥median* | 1.5 (0.7 to 2.4) | 0 (0 to 0.1) | –1.5 | <0.001 | <0.001 |  |
| *Diff. in MDS* | –2.5 | –0.7 |  |  |  | <0.001 |
|  |  |  |  |  |  |  |
| **Vitamin B12** |  |  |  |  |  |  |
| *MDS <median* | 1.0 (0.6 to 1.4) | 1.5 (0.5 to 2.5) | +0.5 | 0.278 | 0.329 |  |
| *MDS ≥median* | 2.4 (1.4 to 3.4) | 0.6 (0.3 to 0.9) | –1.8 | <0.001 | <0.001 |  |
| *Diff. in MDS* | +1.4 | –0.9 |  |  |  | 0.286 |
|  |  |  |  |  |  |  |
| **Vitamin C** |  |  |  |  |  |  |
| *MDS <median* | 4.6 (3.8 to 5.5) | 1.1 (0.2 to 2.0) | –3.5 | <0.001 | <0.001 |  |
| *MDS ≥median* | 1.3 (0.6 to 2.1) | 0.2 (0 to 0.3) | –1.1 | <0.001 | <0.001 |  |
| *Diff. In MDS* | –3.3 | –0.9 |  |  |  | <0.001 |
|  |  |  |  |  |  |  |
| **Vitamin D** |  |  |  |  |  |  |
| *MDS <median* | 96.0 (95.2 to 96.7) | 71.4 (67.6 to 75.2) | –24.6 | <0.001 | <0.001 |  |
| *MDS ≥median* | 92.5 (90.8 to 94.2) | 48.0 (46.2 to 49.8) | –44.5 | <0.001 | <0.001 |  |
| *Diff. in MDS* | –3.3 | –23.4 |  |  |  | <0.001 |
|  |  |  |  |  |  |  |
| **Vitamin E** |  |  |  |  |  |  |
| *MDS <median* | 97.0 (96.3 to 97.6) | 95.8 (94.1 to 97.5) | –1.2 | 0.031 | 0.149 |  |
| *MDS ≥median* | 96.3 (95.1 to 97.6) | 87.3 (86.1 to 88.5) | –9 | <0.001 | <0.001 |  |
| *Diff. in MDS* | –0.7 | –8.5 |  |  |  | <0.001 |
|  |  |  |  |  |  |  |
| **Folic acid** |  |  |  |  |  |  |
| *MDS <median* | 63.0 (61.2 to 64.9) | 36.7 (32.6 to 40.8) | –26.3 | <0.001 | <0.001 |  |
| *MDS ≥median* | 30.4 (27.4 to 33.4) | 9.3 (8.2 to 10.3) | –21.1 | <0.001 | <0.001 |  |
| *Diff. in MDS* | –32.6 | –27.4 |  |  |  | <0.001 |
|  |  |  |  |  |  |  |

Means and IC 95% are expressed in % of participants in the SUN cohort with intakes below Estimated Average Requirement (EAR) stratified by MDS adherence (<median and ≥median). Mediterranean diet (MDS) adherence score range 0-9 points, <median range to 0-3 points and ≥median range to 4-9 points. P for trend was calculated assigning the median value to each quintile and considered the variable as continuous.


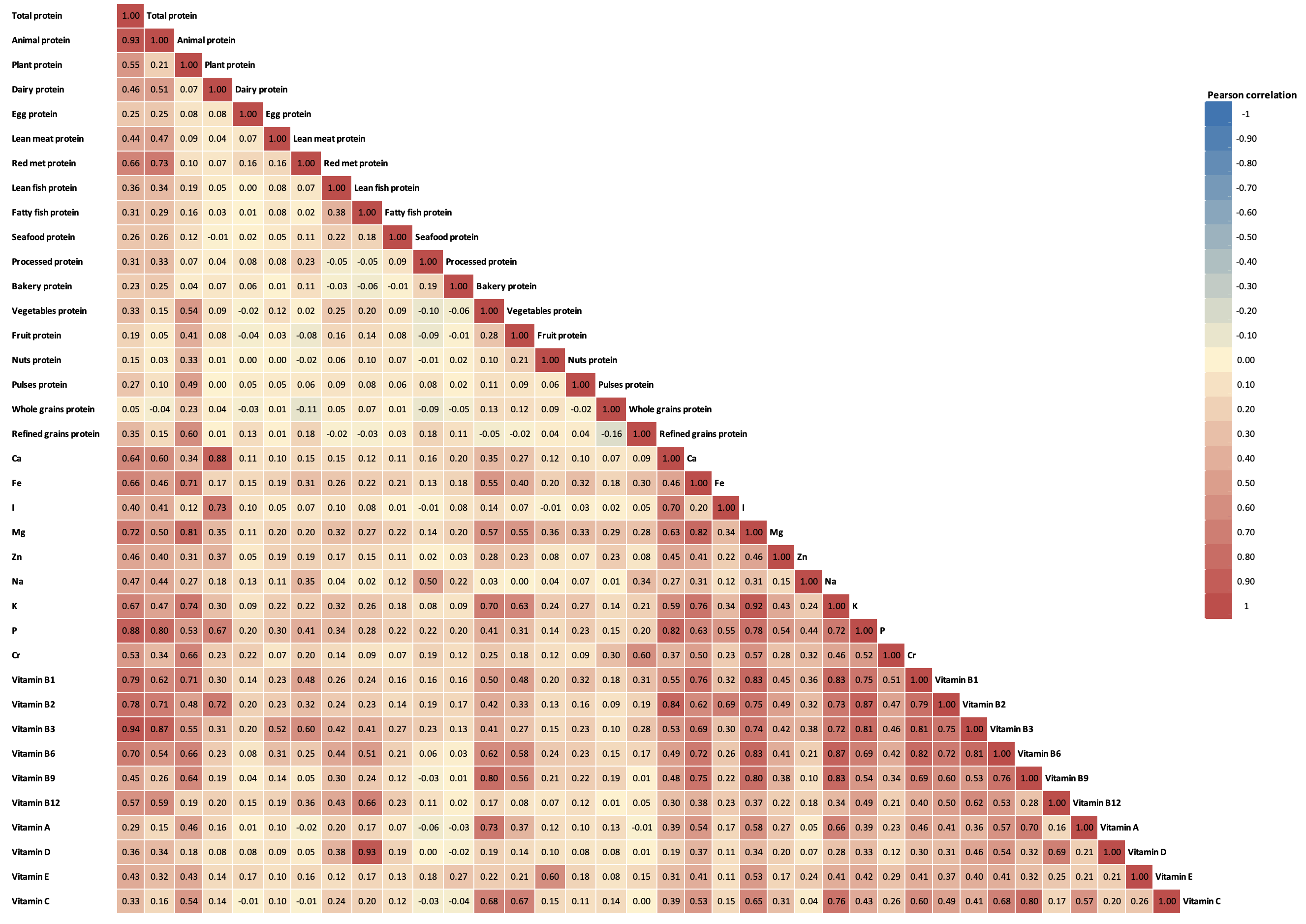


**Supplemental figure 1.** Partial correlation analysis between protein intake by source and micronutrient intake in the SUN cohort participants (N=17,535). Pearson's correlation analysis was used to calculate partial correlation coefficients. The colors blue and red indicate positive and negative correlations, respectively.

**

**Supplemental figure 2.** Comparation of our main analysis with analysis without exclusion the participants with implausible energy intakes and with different exclusions energy criteria (according to percentiles). OR and 95% CI of unmet ≥4 or 8 Dietary Reference Intakes respectively according to PQI in 17,535 participants of the SUN Project. OR were adjusted for age (continuous), sex and education level (graduate, master, doctorate), energy intake (continuous), BMI (continuous), physical activity (MET-h/week), smoking status (no smoking, former, current <15 cig/d, current ≥15 cig/d) and dietary supplement use (yes/no). Numbers are value of hazard ratio. **All models performance a linear trend P value <0.001**


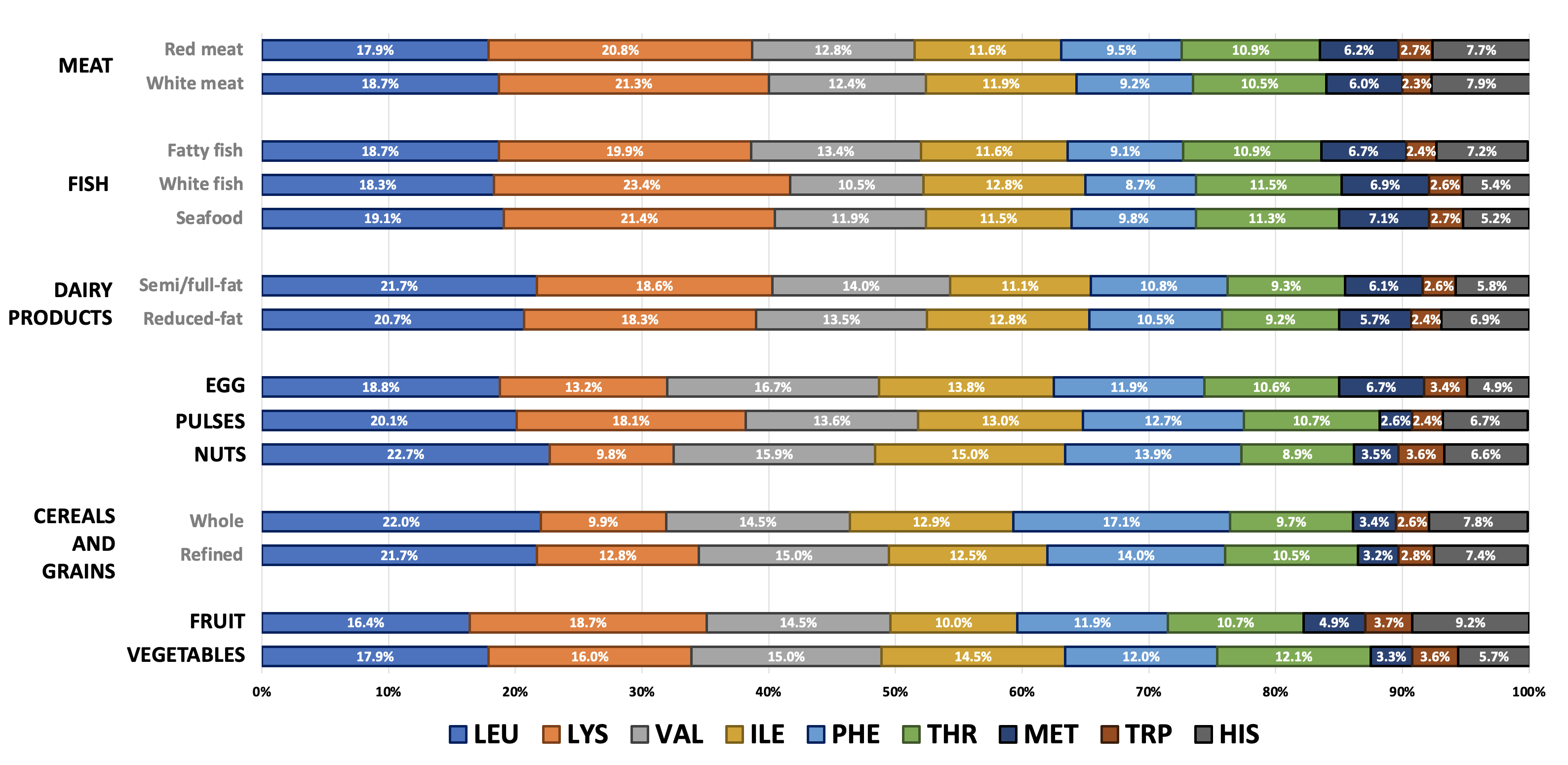


**Supplemental figure 3.** Proportions of essential amino acids intake content across food groups in the SUN cohort participants (N=17,535). Essential amino acids are grouped in descending order of prevalence within food group.
